# Bladder neck size and its association with urinary continence after robot‐assisted radical prostatectomy

**DOI:** 10.1002/bco2.188

**Published:** 2022-09-14

**Authors:** Yasuo Kohjimoto, Masatoshi Higuchi, Shimpei Yamashita, Kazuro Kikkawa, Isao Hara

**Affiliations:** ^1^ Department of Urology Wakayama Medical University Wakayama Japan

**Keywords:** bladder neck, incontinence, prostate cancer, prostatectomy, urethral length

## Abstract

**Objectives:**

This study aims to determine whether bladder neck size (BNS) measured during surgery is associated with urinary continence after robot‐assisted radical prostatectomy.

**Patients and Methods:**

Between June 2015 and March 2019, 365 consecutive eligible patients undergoing robot‐assisted radical prostatectomy were enrolled into a prospective observational cohort study. The primary outcome was patient‐reported urinary continence status at 1, 3, 6, 12 and 24 months postoperatively, with continence defined as 0 pad/day. The primary exposure was BNS (largest diameter) measured intraoperatively just before performance of vesicourethral anastomosis. Other covariates included age, body mass index, NCCN risk category, nerve‐sparing, membranous urethral length measured intraoperatively and weight of the resected specimen.

**Results:**

Well‐preserved neurovascular bundle (bilateral/unilateral/none) was highly correlated with urinary continence status at every point after surgery. No difference could be seen between the group with BNS ≤17 mm and the >17‐mm group at 1, 3 and 6 months after surgery, but there was better urinary rate of continence in narrow BNS group (≤17 mm) at 12 and 24 months after surgery. Multivariate analysis showed both nerve sparing and bladder neck diameter to be independent factors affecting urinary continence at 12 and 24 months after surgery.

**Conclusion:**

Preservation of neurovascular bundles was associated with better urinary continence after surgery. Smaller BNS was associated with better urinary continence in late stages after surgery (12–24 months after surgery).

## 1 INTRODUCTION

Robot‐assisted radical prostatectomy (RARP) has become a standard treatment option for localized prostate cancer. Anatomical approach and improvement of surgical techniques considerably reduced postoperative urinary incontinence, but there are still patients who require urinary pads. The factors affecting urinary continence after radical prostatectomy (RP) have been widely studied. Independent predictive factors for better recovery of urinary continence after RP include younger age, smaller prostate volume and lower body mass index (BMI).^1^ Presence of preoperative overactive bladder was shown to be a negative predictor for recovery of urinary continence after RP.^2^ Surgeon's technical performance has been hypothesized to be linked to functional outcomes in RARP.^3^

Meanwhile, various surgical techniques have been introduced to obtain better urinary continence. Many surgeons adopt posterior reconstruction of the rhabdosphincter, as presented by Rocco et al.,^4^ while Student et al. proposed an advanced reconstruction of vesicourethral support (ARVUS) technique.^5^

From an anatomical point of view, the external urethral sphincter (rhabdosphincter) and internal urethral sphincter play an important role for urinary continence; the function of the external urethral sphincter muscle largely controls urinary continence. Evaluation for bladder neck preservation technique, however, remains controversial.

In this study, we directly measured bladder neck size by ruler during RARP and investigated the relationship between bladder neck size and postoperative urinary continence. Although we applied anterior approach at the bladder neck dissection, bladder neck size varied according to the degree of protrusion of prostate, size of prostate and surgeon's skilfulness. We would like to know the effect of the bladder neck size as a result of surgery on urinary incontinence. We also measured urethral length by ruler during RARP. Urethral length is usually measured by preoperative MRI. However, we would like to know the effect of the urethral length as a result of surgery on urinary incontinence.

## 2 PATIENTS AND METHODS

### 2.1 Study population

This prospective observational cohort study enrolled 365 consecutive eligible patients undergoing RARP between June 2015 and March 2019 at our hospital. This study was approved by the Wakayama Medical University Institutional Review Board (IRB# 1670). RARP was performed by three surgeons according the standard techniques, as described previously.^6^ These three surgeons experienced 64, 63 and 58 cases of RARP before this study. At the bladder neck dissection, anterior approach was applied.^7^ To avoid positive surgical margin, we did not intend to perform very aggressive BNP. Consequently, bladder neck size became larger when the middle lobe of the prostate was protruded, or tumour lesion was suspected at the base of the prostate by MRI. In these cases, bladder neck reefing was added after urethra–bladder anastomosis. Surgical procedures that may affect postoperative urinary continence, such as posterior musculofascial reconstruction, urethro‐bladder anastomosis and periurethral suspension stitch, were performed in the same manner in all cases. Briefly, posterior wall reconstruction was performed as reported by Rocco et al.,^4^ and urethra–bladder anastomosis was performed by continuous suturing using two barbed threads (3‐0 V‐LocTM). The periurethral retropubic stitch was passed from periurethral tissue to the periostium on the pubic bone using a monofilament polyglytone suture on a CT‐1 needle.^8^

The indication of neurovascular bundle preservation was determined by oncological aspects. We abandoned neurovascular bundle preservation in the case of (1) palpable induration by digital rectal examination, (2) PI‐RADS 4–5 lesions were pointed out by preoperative MRI and (3) number of positive biopsy core ≥3. Therefore, no relationship between preoperative IIEF‐5 score and the status of neurovascular bundle preservation was found.

### 2.2 Patient reported outcome

To evaluate the status of urinary incontinence, Expanded Prostate Cancer Index Composite (EPIC) was used to report patient outcome. The Japanese version of EPIC was purchased from iHope International (Kyoto, Japan).^9^ EPIC questionnaire was handed to each patient before surgery and then periodically mailed to each patient postoperatively, (1, 3, 6, 12 and 24 months after operation) to be returned to our hospital. Urinary continence was defined as 0 pad/day.

### 2.3 Measurement of bladder neck size and urethral length

Bladder neck size and urethral length were measured intraoperatively. Bladder neck was measured in millimetres by ruler inserted through an assistant port just before vesicourethral anastomosis. Urethral length was also measured from the dorsal vein complex stump by ruler after cutting the urethra.

### 2.4 Primary outcome, primary exposure and covariates

The primary outcome was patient‐reported urinary continence status at 1, 3, 6, 12 and 24 months postoperatively, with continence defined as 0 pad/day. The primary exposure was BNS (largest diameter) measured intraoperatively just before vesicourethral anastomosis. Other covariates included age, body mass index, NCCN risk category, nerve‐sparing, membranous urethral length measured intraoperatively and weight of resected specimen.

### 2.5 Statistical analyses

The relationship between continence status and BNS was evaluated using chi square test and multivariate logistic regression analysis. Data analyses were conducted using the statistical software JMP Pro 12 (SAS Institute, Cary, NC). All *P* values were two‐tailed, and *P* < 0.05 was defined as statistically significant.

## 3 RESULTS

### 3.1 Patient characteristics

Patient characteristics are shown in Table 1. The median bladder neck size and urethral length from dorsal vein complex stump were 17 and 15 mm, respectively. The response rates to EPIC questionnaire before operation and then 1, 3, 6, 12 and 24 months after surgery were 98.4%, 98.4%, 96.5%, 90.9%, 89.3% and 80%, respectively.

**TABLE 1:** Patient characteristics (*N* = 365)

Age, years	69 (65–72)
BMI, kg/m^2^	23.9 (21.9–26.0)
PSA, ng/ml	8.1 (6.0–11.4)
Biopsy Gleason grade, *n* (%)	
Group 1	87 (23.9)
Group 2	87 (23.9)
Group 3	81 (22.3)
Group 4	79 (21.7)
Group 5	30 (8.2)
cT stage, *n* (%)	
cT1c	68 (18.6)
cT2	264 (72.3)
cT3	33 (9.0)
NCCN risk category, *n* (%)	
Low	48 (13.2)
Intermediate	184 (50.4)
High	133 (36.4)
Preoperative ADT, *n* (%)	9 (2.5)
Nerve‐sparing, *n* (%)	
None	74 (20.3)
Unilateral	199 (54.5)
Bilateral	92 (25.2)
Urethral length, mm	
From urogenital diaphragm	22 (19–25)
From DVC stump	15 (13–17)
Bladder neck diameter, mm	17 (14–22)
Weight of resected specimen, g	43 (36–54)

Abbreviations: ADT, androgen deprivation therapy; BMI, body mass index; DVC, dorsal vein complex; PSA, prostate‐specific antigen.

### 3.2 Urinary continence rate

**FIGURE 1:**
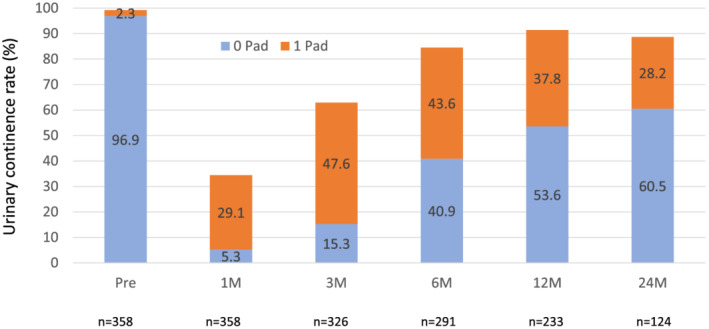
Overall urinary continence rate. Urinary continence was defined as 0 pad/day.

Although Figure 1 shows overall urinary continence rate according to the number of pad per day (0 or 1 pad/day), we defined urinary continence was defined as only 0 pad/day. Therefore, urinary continence rate at 24 months after surgery was 60.5%, which was worse than when urinary continence was defined as 0–1 pad/day (88.7%).

**FIGURE 2:**
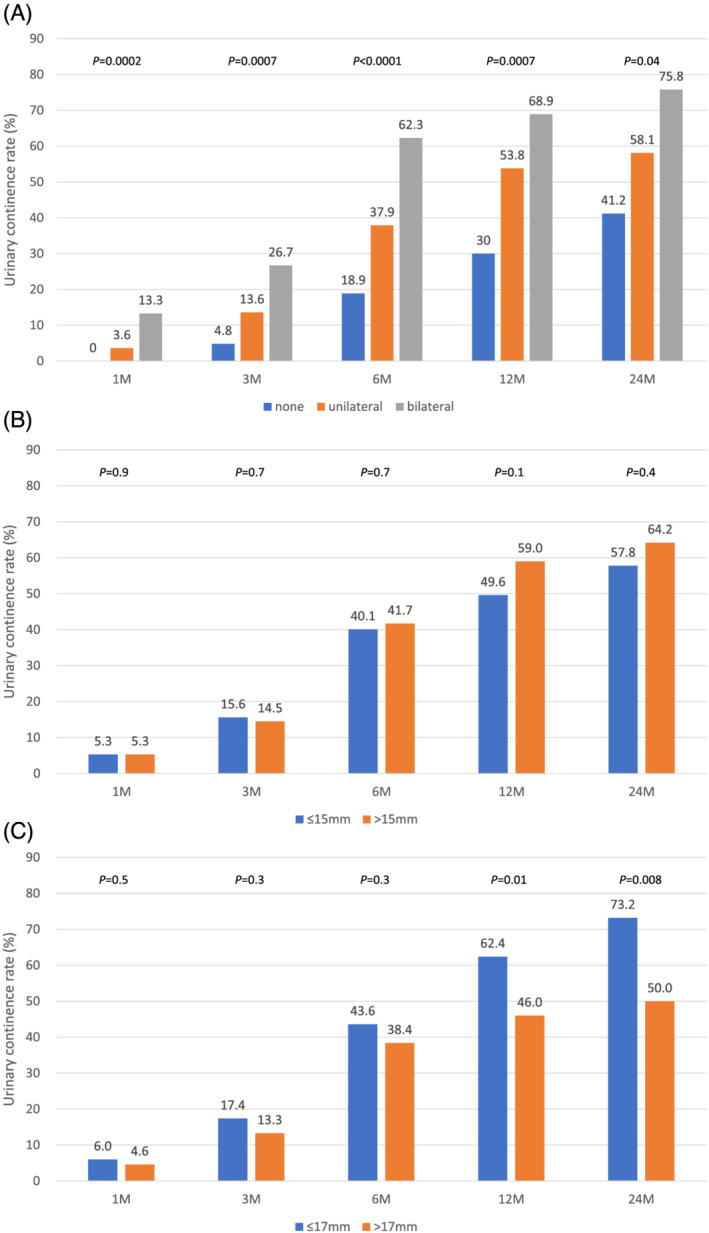
(A) Urinary continence rate according to the status of nerve sparing (blue: not preserved; orange: unilateral side was preserved; grey: bilateral sides were preserved). (B) Urinary continence rate according to urethral length (blue: urethral length ≤15 mm; orange: urethral length >15 mm). (C) Urinary continence rate according to bladder neck diameter (blue: bladder neck diameter ≤17 mm; orange: bladder neck diameter >17 mm)

Figure 2A shows the urinary continence rate according to the nerve sparing status. Well‐preserved neurovascular bundle (bilateral/unilateral/none) was clearly shown to be highly correlated with urinary continence status at every point after surgery.

Figure 2B shows the urinary continence rate according to the remaining urethral length. There seems to be tendency of longer remaining urethra being associated with better urinary continence at 12 and 24 months after surgery, although without significant difference.

Figure 2C shows the rate of urinary continence according to the bladder neck diameter. No difference could be seen between bladder neck size ≤17‐ and >17‐mm groups at 1, 3 and 6 months after surgery, but better urinary continence rate was observed in narrow bladder neck size group (≤17 mm) at 12 and 24 months after surgery.

**FIGURE 3:**
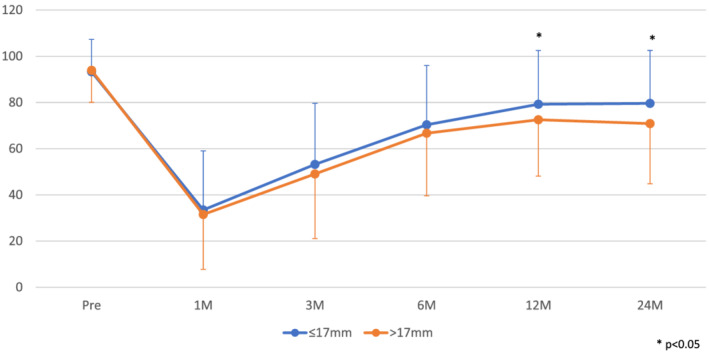
Expanded Prostate Cancer Index Composite (EPIC) Urinary Incontinence score according to bladder neck diameter

Figure 3 shows the EPIC Urinary Incontinence score according to bladder neck diameter. No difference could be seen between bladder neck size ≤17‐ and >17‐mm groups at 1, 3 and 6 months after surgery, but better EPIC Urinary Incontinence score was observed in narrow bladder neck size group (≤17 mm) at 12 and 24 months after surgery. This result was concordance with the rate of urinary continence shown in Figure 2C.

We performed multivariate analysis on the factors affecting urinary continence status at 12 and 24 months after surgery (Table 2). Nerve sparing status, urethral length and bladder neck size were found to be independent factors affecting urinary continence status at 12 months after surgery. At 24 months after surgery, only nerve sparing status and bladder neck size remained as independent factors affecting urinary continence status.

**TABLE 2:** Predicting factors for urinary continence at 12 and 24 months: Multivariate analysis

	12 months			24 months		
	OR	95% CI	*p*	OR	95% CI	*p*
Age (≤69/>69 years)	0.84	0.43–1.61	0.6	0.48	0.28–1.97	0.5
BMI (≤23.9/>23.9 kg/m^2^)	1.31	0.70–2.42	0.38	0.74	0.28–1.97	0.5
NCCN risk category						
Low	Reference			Reference		
Intermediate	0.45	0.17–1.19	0.1	0.70	0.18–2.76	0.6
High	0.37	0.13–1.07	0.06	0.65	0.14–2.93	0.5
Weight of resected specimen (≤43/>43 g)	1.35	0.70–2.59	0.3	1.62	0.61–4.31	0.3
Nerve‐sparing						
None	Reference			Reference		
Unilateral	3.85	1.57–9.45	0.003	4.12	0.96–17.54	0.05
Bilateral	5.18	1.77–15.09	0.002	9.95	1.58–62.36	0.01
Urethral length (>15/≤15 mm)	2.23	1.13–4.38	0.01	2.04	0.76–5.45	0.15
Bladder neck diameter (≤17/>17 mm)	3.38	1.70–6.71	0.0005	3.68	1.31–10.34	0.01
IIEF						
5–7 severe	Reference			Reference		
8–11 moderate	0.29	0.08–1.00	0.05	0.66	0.17–2.55	0.5
12–16 mild to moderate	2.08	0.69–6.29	0.1	3.83	0.75–19.46	0.1
17–21 mild	0.95	0.39–2.28	0.9	6.10	1.16–32.10	0.03
22–25 No ED	0.48	0.14–1.66	0.2	0.37	0.08–1.71	0.20

Abbreviations: BMI, body mass index; ED, erectile dysfunction; IIEF, International Index of Erectile Function.

## 4 DISCUSSION

Urinary incontinence after RP can have substantial impact on the patient's postoperative quality of life. The mechanisms directly involved in urinary continence are the smooth muscle (inner sphincter) of the bladder neck and the striated muscle (rhabdosphincter) present in the membranous urethra. Of these, the rhabdosphincter seems to play a major role in urinary continence. Shorter length of resected membranous urethra measured in haematoxylin and eosin sections at the apical margin of prostate specimens showed significantly better urinary continence after RP.^10^ Moreover, a meta‐analysis of the membranous urethral length evaluated by preoperative MRI showed that longer membranous urethra was associated with better urinary continence after RP in all papers that reported the hazard ratio.^11^

Conversely, the purpose of the bladder neck‐preserving technique is to improve urinary incontinence by preserving the internal sphincter. Although meta‐analysis has concluded that the bladder neck‐preserving technique improves urinary continence after RP,^12^ several papers have reported to the contrary.^13^, ^14^, ^15^ Bladder neck‐preserving technique does not therefore appear to affect postoperative urinary continence as much as the external urethral sphincter. It seems that the more perfectly preserved the bladder neck, the smaller the internal urethral diameter, but only one paper actually measured the internal urethral diameter and investigated its association with postoperative urinary incontinence.^16^ No association has been shown between the internal urethral diameter actually measured during surgery and postoperative urinary incontinence. Although there was similarity in our research to that by Tyson et al., we obtained different results; in our study, there was correlation between bladder neck size measured during surgery and urinary continence in the late postoperative period (12–24 months). We speculated the reasons for acquisition of different results. First, we investigated the urinary continence rate over a long period (from 1 to 24 months after surgery), while Tyson et al. investigated only the early period after surgery (6 and 12 weeks after surgery). No statistically significant differences were observed in the early stages after surgery in our study either, so it might be possible that significant difference would also have been observed in late stages after surgery in Tyson's study. Second, we did not pursue aggressive bladder neck preservation, and the extent to which the bladder neck was preserved varied depending on the preference of the surgeon and the patient's condition. There were thus two possible patterns of increased bladder neck size: one is when the bladder neck is transected on the bladder side of the appropriate line due to the surgeon's inexperience, the other is due to the patient's anatomical factors (patients with marked middle lobe protrusion of the prostate or after transurethral resection of the prostate). The surgeon's different attitude towards bladder neck dissection might be another reasons for differing results. A third reason for different results in the two studies is perhaps the definition of urinary continence being different between them; we defined urinary continence as the use of no pads in our study, while Tyson et al. presented EPIC incontinence score as degree of urinary continence. There were also differing objective variables between the studies.

In this study, urethral length measured during surgery was not associated with postoperative urinary continence, although it was an independent factor affecting urinary continence only at 12 months after surgery. We previously reported that the length of resected membranous urethra measured by resected specimen was a predictor of urinary incontinence after RP,^10^ so our current results seem to be contradictory. We assume that the method of measuring urethral length during surgery was inappropriate. We measured urethral length just before cutting urethra, so the prostate itself was pulled toward head by Prograsp forceps and so the measured urethral length was influenced by the degree of traction.

This study has some limitations. Several different operators performed surgery in our study, so there was variation in skill and attitude towards RARP. With accumulation of more cases, it might be interesting to perform a similar study with consideration of each surgeon individually.

In conclusion, preservation of neurovascular bundles was significantly associated with better urinary continence after surgery. Smaller bladder neck size was significantly associated with better urinary continence in later stages (12–24 months) after surgery.

## CONFLICT OF INTEREST

No conflict of interest.

## AUTHOR CONTRIBUTIONS

YK made substantial contributions to the conception of the work. MH made significant contributions to collecting the data and statistical analysis. SY and KK made contributions to the interpretation of the data. IH drafted the original manuscript. All authors substantially contributed to the revision of the manuscript drafts and have approved the final version of the manuscript.

## References

[1] Kim JJ , Ha YS , Kim JH , Jeon SS , Lee DH , Kim WJ , et al. Independent predictors of recovery of continence 3 months after robot‐assisted laparoscopic radical prostatectomy. J Endourol. 2012;26(10):1290–5. 22651546 10.1089/end.2012.0117 PMC3466068

[2] Yamada Y , Fujimura T , Fukuhara H , Sugihara T , Miyazaki H , Nakagawa T , et al. Overactive bladder is a negative predictor of achieving continence after robot‐assisted radical prostatectomy. Int J Urol. 2017;24(10):749–56. 28697538 10.1111/iju.13411

[3] Goldenberg MG , Goldenberg L , Grantcharov TP . Surgeon performance predicts early continence after robot‐assisted radical prostatectomy. J Endourol. 2017;31(9):858–63. 28557582 10.1089/end.2017.0284

[4] Rocco B , Gregori A , Stener S , Santoro L , Bozzola A , Galli S , et al. Posterior reconstruction of the rhabdosphincter allows a rapid recovery of continence after transperitoneal videolaparoscopic radical prostatectomy. Eur Urol. 2007;51(4):996–1003. 17079070 10.1016/j.eururo.2006.10.014

[5] Student V Jr , Vidlar A , Grepl M , Hartmann I , Buresova E , Student V . Advanced Reconstruction of Vesicourethral Support (ARVUS) during robot‐assisted radical prostatectomy: One‐year functional outcomes in a two‐group randomised controlled trial. Eur Urol. 2017;71(5):822–30. 27283216 10.1016/j.eururo.2016.05.032

[6] Koike H , Kohjimoto Y , Iba A , Kikkawa K , Yamashita S , Iguchi T , et al. Health‐related quality of life after robot‐assisted radical prostatectomy compared with laparoscopic radical prostatectomy. J Robot Surg. 2017. 10.1007/s11701-016-0659-8 28130703

[7] Kojima Y , Takahashi N , Haga N , Nomiya M , Yanagida T , Ishibashi K , et al. Urinary incontinence after robot‐assisted radical prostatectomy: Pathophysiology and intraoperative techniques to improve surgical outcome. Int J Urol. 2013;20(11):1052–63. 23841851 10.1111/iju.12214

[8] Patel VR , Coelho RF , Palmer KJ , Rocco B . Periurethral suspension stitch during robot‐assisted laparoscopic radical prostatectomy: Description of the technique and continence outcomes. Eur Urol. 2009;56(3):472–8. 19560260 10.1016/j.eururo.2009.06.007

[9] Takegami M , Suzukamo Y , Sanda MG , Kamoto T , Namiki S , Arai Y , et al. The Japanese translation and cultural adaptation of expanded prostate Cancer index composite (EPIC). Nihon Hinyokika Gakkai Zasshi the Japanese Journal of Urology. 2005;96(7):657–69. 16363651 10.5980/jpnjurol1989.96.657

[10] Kohjimoto Y , Yamashita S , Kikkawa K , Iba A , Matsumura N , Hara I . The Association of Length of the resected membranous urethra with urinary incontinence after radical prostatectomy. Urol J. 2020;17(2):146–51. 30882170 10.22037/uj.v0i0.4753

[11] Mungovan SF , Sandhu JS , Akin O , Smart NA , Graham PL , Patel MI . Preoperative membranous urethral length measurement and continence recovery following radical prostatectomy: A systematic review and Meta‐analysis. Eur Urol. 2017;71(3):368–78. 27394644 10.1016/j.eururo.2016.06.023 PMC5600894

[12] Ma X , Tang K , Yang C , Wu G , Xu N , Wang M , et al. Bladder neck preservation improves time to continence after radical prostatectomy: A systematic review and meta‐analysis. Oncotarget. 2016;7(41):67463–75. 27634899 10.18632/oncotarget.11997 PMC5341889

[13] Poon M , Ruckle H , Bamshad BR , Tsai C , Webster R , Lui P . Radical retropubic prostatectomy: Bladder neck preservation versus reconstruction. J Urol. 2000;163(1):194–8. 10604345 10.1016/s0022-5347(05)68003-2

[14] Srougi M , Nesrallah LJ , Kauffmann JR , Nesrallah A , Leite KR . Urinary continence and pathological outcome after bladder neck preservation during radical retropubic prostatectomy: A randomized prospective trial. J Urol. 2001;165(3):815–8. 11176476

[15] Wei JT , Dunn RL , Marcovich R , Montie JE , Sanda MG . Prospective assessment of patient reported urinary continence after radical prostatectomy. J Urol. 2000;164(3 Pt 1):744–8. 10953138 10.1097/00005392-200009010-00029

[16] Tyson MD 2nd , Ark J , Gregg JR , Johnsen NV , Kappa SF , Lee DJ , et al. The null effect of bladder neck size on incontinence outcomes after radical prostatectomy. J Urol. 2017;198(6):1404–8. 28655528 10.1016/j.juro.2017.06.084 PMC5693618

